# Efficacy of interferential low frequency therapy for elderly wet overactive bladder patients

**DOI:** 10.4103/0970-1591.40611

**Published:** 2008

**Authors:** Hitoshi Oh-oka

**Affiliations:** Department of Urology, Independent Administrative Institution, National Hospital organization, Kobe Medical Center, Kobe, Japan

**Keywords:** Elderly wet OAB, interferential low frequency therapy, QOL

## Abstract

**Objectives:**

Examining the clinical efficacy on the interferential low frequency therapy (IF) for elderly nonneurogenic (idiopathic) overactive bladder patients with urgent urinary incontinence (wet OAB) prospectively, for whom anticholinergics were not effective.

**Materials and Methods:**

Subjects are elderly 80 patients (69-78, median age 72.0) with urinary incontinence, who are clinically diagnosed with wet OAB without urodynamics (pressure/flow study). For 3 months, they were administered anticholinergics (propiverine hydrochloride 20 mg/once per day in the morning), but all their quality of life (QOL) score were 4 or over due to poor control of storage symptoms and urinary incontinence. We selected patients for whom anticholinergics were not effective (above-mentioned 80 patients) and they were provided with IF alone for 3 months with informed consent. Before and after IF, the followings were examined. (1) frequency of IF treatment required to show optimal effects, (2) average weekly frequency of incontinence, (3) 60-min pad test, (4) frequency and voided volume in the daytime and nighttime, (5) fluid intake volume, (6) International Prostate Symptom Score, quality of life score, (7) Uroflowmetry, (8) postvoid residual urine volume, (9) specific gravity of urine, (10) average hours spent outdoors, (11) average radius of action and activities of daily life score, (12) standing blood pressure (BP) and heart rate, (13) clinical laboratory findings, (14) adverse events, (15) plasma osmotic pressure (OP), and (16) Brain natriuretic peptide (BNP).

**Results:**

(1) The patients showed improvements for eight treatments (median). Improvement was observed in the followings: (2), (3), (4) voiding frequency, (6), (7) voided volume, maximum and average flow rate, (10), (11), (12) BP, (15) OP, and (16).

**Conclusions:**

The IF has safe and better effects than anticholinergics on the elderly wet OAB patients.

## INTRODUCTION

Interferential therapy (IF) utilizing effects of low frequency electrical stimulation was first conducted on the lower urinary tract in the treatment of urinary incontinence by McQuire.[1] The utility of this therapy for conditions such as urinary frequency, urge incontinence, and stress incontinence was subsequently reported by researchers including Dougall, [2] Laycock and Green,[3] and Switzer and Hendriks.[4] Incidence of OAB increases with age.[5] OAB significantly lowers quality of life.[6] Patients also suffer with more depression, poor quality of sleep.[7] Recent study showed that some elderly patients with OAB had high-plasma osmolality and brain natriuretic peptide (BNP) levels despite low urine specific gravity.[8] Elevated BNP levels (50 pg/ml or higher) suggest the possibility of concomitant cardiovascular diseases and require a thorough examination[9] because of affecting quality of life (QOL), activity of daily life (ADL), and nocturia. Here I report on the effects of IF on overactive bladder with incontinence (“wet OAB”) in the elderly which is poorly controlled by anticholinergic treatment, along with the examination of cardiac function, hemoconcentration, and urine concentration.

## MATERIALS AND METHODS

The study subjects were 80 patients aged 65 years or over (40 males and 40 females, 69-78, median age 72.0) with nonneurogenic (idiopathic) “wet OAB” who are clinically diagnosed without urodynamics (pressure/flow study) and no medical history of extra-urological conditions including clinically evident cardiac diseases. None was experiencing serious complications nor undergoing any medical or other therapy that would possibly influence their micturition state. Electrocardiography, echocardiography, and other examinations conducted at the Department of Cardiovascular Internal Medicine of the author's hospital demonstrated the absence of conditions requiring clinical treatment in those patients to exclude potential cardiovascular diseases previously. These patients were given anticholinergic treatment (propiverine hydrochloride 20 mg/day) once a day in the morning for 3 months or longer, with poor control of storage symptoms and urinary incontinence the QOL index scoring at a four (mostly dissatisfied) or more as revealed in the posttreatment interview. All the patients offered cancellation of anticholinergics and hope alternative noninvasive therapy for further improvement of their symptoms. After consultation and written informed consent about IF (including full explanation about weak evidence compared with anticholinergics for OAB treatment) was obtained from each participating patient, a washout period of at least 1 week instituted following the anticholinergic treatment, a 3-month IF alone was performed at 20 Hz and 20 mA (Uromaster^®^, Nihon Medix Co. Ltd, Matsudo, Chiba, Japan), using surface electrodes placed at four sites in the lower abdomen and lower buttocks. A 20-min treatment session was conducted twice a week for the first 3 weeks and once every 2 weeks thereafter. Prior to treatment initiation, the patients were telephoned from the hospital once to twice per week to give a brief information, apart from a voiding diary, to their urologists on their subjective and objective findings regarding wet OAB (e.g., volume of leakage, frequency and degree of urinary urgency, and voiding frequency) during the interview held every 2 weeks because of inspecting validity of IF continuation (weak evidence of efficacy of IF for OAB). The following data were compared before and after IF: (1) number of treatment sessions required for improvement to be noted in the assessment made in interviews concerning on subjective symptoms, (2) average weekly frequency of incontinence, (3) 60-min pad test results, (4) frequency and volume of daytime and nighttime voiding, (5) volume of water intake, (6) International Prostate Symptom Score (IPSS) and QOL index score, (7) uroflowmetry test values (voiding time, single voided volume, maximum flow rate [Q_max_], and average flow rate [Q_ave_]), (8) postvoid residual urine volume (PVR), (9) specific gravity of urine, (10) average hours spent outdoors in the day, (11) one-day average radius of action and activities of daily life scale score, (12) standing blood pressure (BP) and heart rate (HR), (13) clinical laboratory findings, (14) IF-related adverse events, (15) plasma osmotic pressure (OP) and (16) BNP. The conduct of this study was approved by the Kobe Medical Center Ethics Committee. No behavioral therapy, including water intake instructions, was provided during the study period. Patients were asked to record in their voiding diary 5-day data including data for two successive days. The median was adopted as study data for voiding frequency, single voided volume, and water intake volume. The median of three measurements was adopted for uroflowmetry test data, PVR, BP and HR; PVR was conducted by catheterization at three different times during the day. Changes in hours spent outdoors, radius of action, and ADL scale score were used to assess the patients' lifestyle. As the average hours spent outdoors in one day, the median of daily hours spent outdoors in a 14-day period was adopted. As the one-day average radius of action, the median of the farthest distance traveled from home over a 14-day period was adopted; the distance was measured roughly on a map. For the ADL scale score, a 10-grade visual analogue scale (VAS) from 0 to 9 originally developed at Kobe Medical Center, was also used (0, ADL unaffected; 3, slightly affected; 6, moderately affected; and 9, highly affected). In statistical analyses, paired *t*-test was used for the comparison of paired two samples and Wilcoxon signed rank test for the comparison of ordinal data from two independent samples; *P* < 0.05 was considered statistically significant.

## RESULTS

Therapeutic effects of IF: Self-reported improvement in subjective symptoms appeared after 5-14 (median, 8) treatment sessions; the improved condition lasted for 3 months without reversal. All patients completed the scheduled course of treatment.

Changes in one-week average frequency of incontinence and 60-min pad test results. Both parameters improved significantly. The frequency of incontinence decreased from 13.3 ± 5.2 (mean ± standard deviation) times/week at pre-IF to 3.6 ± 3.5 times/week in a 3-month period on IF (*P* < 0.0001) and the weight of pad in the 60-min pad test, from 17.5 ± 2.1 to 3.1 ± 2.1 g (*P* < 0.0001).

Changes in the frequency and volume of daytime and nighttime voiding: The voiding frequency decreased significantly in both the daytime, from 8.3 ± 2.4 to 7.0 ± 1.8 times (*P* < 0.0001) and the nighttime, from 1.8 ± 1.0 to 1.4 ± 1.0 times (*P* = 0.0004). The daytime voided volume showed an increasing tendency, from 1199 ± 230 to 1220 ± 320 ml while the nighttime volume showed a decreasing tendency, from 514 ± 185 to 464 ± 157 ml; neither changes were significant.

Change in daily water intake volume: An increasing tendency without statistical significance, from 1270 ± 372 to 1332 ± 289 ml, was noted.

**Figure 1:**
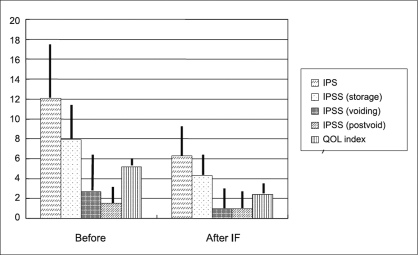
Changes of IPSS, QOL score. Storage; storage symptoms, voiding; voiding symptoms, postvoid; postvoid symptoms

Changes in IPSS and QOL index score [Figure 1]. The total IPSS improved with statistical significance (*P* < 0.0001) from 12.1 ± 5.3 to 6.3 ± 3.3. When the IPSS was subdivided into symptom scores related to storage, voiding and postvoiding, all subdivision symptom scores showed significant improvement: storage, from 7.9 ± 3.6 to 4.3 ± 2.5 (*P* < 0.0001); voiding, from 2.7 ± 3.8 to 1.0 ± 2.2 (*P* < 0.0001); and postvoiding, from 1.5 ± 1.9 to 1.0 ± 1.6 (*P* = 0.0028). The QOL index score decreased by half from 5.2 ± 0.8 to 2.4 ± 1.1 (*P* < 0.0001).

**Figure 2:**
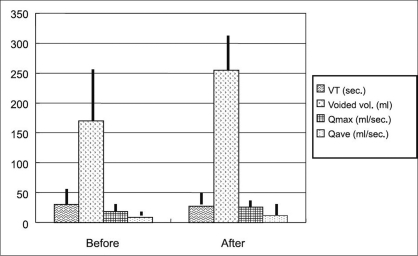
Change in the uroflowmetry test values. VT; voiding time, voided vol.; voided volume, Q_max_; maximum flow rate, Q_ave_; average flow rate

Change in the uroflowmetry test values [Figure 2]. Voiding time did not differ significantly between pre- and post- treatment. The single voided volume improved from 170.2 ± 84.8 to 254.2 ± 60.6 ml (P < 0.0001). Both Q_max_ (from 18.1 ± 6.8 to 25.7 ± 6.6 ml/s) and Q_ave_ (from 8.9 ± 4.1 to 12.1 ± 3.5 ml/s) also improved (P < 0.0001 each).

Change in PVR: The change (from 17.5 ± 24.0 to 14.5 ± 23.1 ml) was considered nonsignificant.

Change in specific gravity of urine: A decreasing trend from 1.019 ± 0.007 to 1.016 ± 0.006 was exhibited, with no statistical significance.

**Figure 3:**
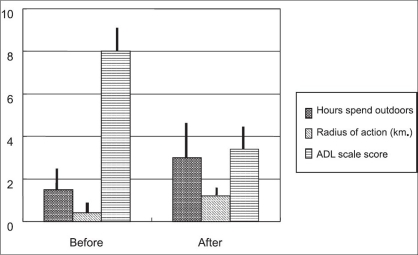
Changes in lifestyle. Hours spent outdoors; the average hours spent outdoors in one day, radius of action; the one-day average radius of action

Changes in lifestyle [Figure 3]. The average hours spent outdoors in one day increased from 1.5 ± 1.3 to 3.0 ± 1.4 hours (*P* < 0.0001), the one-day average radius of action increased from 400 ± 300 to 1200 ± 500 m (*P* < 0.0001) and the ADL scale score decreased from 8.0 ± 1.2 to 3.4 ± 1.5 (*P* < 0.0001); all these improvements were significant. Interviews showed that all patients experienced increases in their amount of outdoor activities, time spent for shopping and hobbies and time spent with their friends and close relatives.

Changing in standing BP, HR and clinical laboratory findings: Both systolic and diastolic BP decreased significantly, from 132.9 ± 8.6 to 127.1 ± 7.4 mm Hg and from 79.7 ± 8.1 to 74.7 ± 8.2 mm Hg, respectively. The change in HR from 73.5 ± 6.8 to 73.0 ± 5.4 bpm was not significant and neither were the changes in laboratory test values.

Adverse events: As an IF-related event reported was a sensation of skin stimulation at the time when current was applied to the electrode surface in the initial treatment session. No such adverse events occurred that were deemed clinically significant, including skin stimulation at the site of electrode placement.

Changes in plasma OP and BNP: Plasma OP increased significantly (*P* < 0.0001) from 295.1 ± 7.8 to 297.8 ± 3.6 mOsm/l while BNP decreased significantly (*P* < 0.0001) from 41.3 ± 38.7 to 19.2 ± 11.1 pg/ml.

## DISCUSSION

Propiverine hydrochloride is presumed to ease OAB by inhibiting afferent transmission of information, undertaken by acetylcholine released from the urothelium via bladder distension stimuli, on bladder tension development control during urine pooling and bladder distension (antimuscarinic action)[10] and by reducing the contraction of bladder smooth muscles (calcium antagonistic action). On the other hand, the possible mechanisms of IF include (1) inhibition of efferent activities of the pelvic nerve through the somatosensory nerve stimulation in the pudendal region (action on the micturition center in the brainstem and the spinal cord),[11,12] (2) increasing the pelvic blood flow,[13] and (3) improving the urine pooling function[14] of the bladder and urinary tract through the sympathetic nerve (hypogastric nerve). Of these, mechanism (1) appears to be mainstream. Although the present study was not of a placebo-controlled or blinded design, it is not deemed reasonable to attribute improvements observed in various parameters solely to placebo effect and electrical stimulation of the skin surface. It is possible that the improvements in frequent urination, urgency, and incontinence were brought about by a mechanism different from that of propiverine hydrochloride, i.e., inhibition by IF of efferent activities of the pelvic nerve. The present study subjects, elderly patients with urinary incontinence or so-called “wet OAB,” did not exhibit a tendency of excessive water intake. Pre-IF plasma OP was high at 295.1 mOsm/l and pre-IF daily water intake was moderate at 1270 ml. Information obtained in the interviews clearly revealed the patients' intention to avoid water intake in an attempt to control incontinence. At post-IF, the frequency of incontinence decreased to about 1/4 and the volume of leakage to about 1/6 of the respective pretreatment levels, together with increase in Q_max_ and single voided volume and decrease in frequency and in urgency. In interviews held during the study, all patients mentioned that their lifestyle became closer to what they wanted it to be thanks to the more favorable state of QOL and ADL resulting from improved “wet OAB” symptoms (average hours spent outdoors roughly doubled and average range of action roughly tripled). This is in agreement with improvements achieved in the QOL index and the ADL scale scores. In addition, relatively early manifestation of improvement in subjective symptoms (within about 7 weeks after IF initiation) appears to prompt patients to increase the amount of suggested exercises (including that of the pelvic floor muscles), contributing to even larger therapeutic effects of IF. Additional advantages include: the absence of complications such as increased frequency and (nighttime) voided volume resulting from greater water intake to counter dry mouth, which is an adverse event observed with the use of anticholinergics including propiverine hydrochloride; and satisfactory compliance that can be achieved in patients who are not indicated for oral treatment or are incapable of oral ingestion.

The present study demonstrated that even in the absence of clinically evident cardiac disorders, plasma OP and BNP levels can be high and that such levels are lowered in a relatively short period of time. The IF can improve clinical symptoms of “wet OAB” with relatively rapidly and thereby can improve ADL levels, as represented by the greater radius of action accompanying symptom amelioration, together with BNP level decrease, implying a reduced load on left ventricular function. These features suggest that IF is a favorable treatment for the elderly.

On the other hand, urine osmotic pressure was isotonic in these patients; although plasma OP significantly increased, the specific gravity of urine did not respond to the change. This indicates impaired urine concentration capacity and decreased cardiac (left ventricular) function in association with advanced age.[15] Given the above results, it should be fully recognized that in elderly patients plasma OP and BNP can be high even when the specific gravity (osmotic pressure) of urine is low and water intake control and related instructions should be considered when water intake restriction or diuretic use is chosen to treat frequent urination (nocturia), giving sufficient consideration to circulating blood volume and cardiac (left ventricular) function. After the present study was completed, prolongation of treatment intervals or discontinuation where possible or therapy change was scheduled to be implemented; however, currently, i.e., about 1 year after study completion, all 80 patients are still undergoing IF every 2 weeks at their request. This fact also suggests satisfactory patient compliance to IF. Combining IF with daily life guidance, including water intake instructions or with behavioral therapy may bring about greater clinical effects. Furthermore, a combination of IF, behavioral therapy, and anticholinergic medication may provide a multidisciplinary approach to the treatment of “wet OAB”.
